# Genetic Connectivity of the Sky Emperor, *Lethrinus mahsena* Populations Across a Gradient of Exploitation Rates in Coastal Kenya

**DOI:** 10.3389/fgene.2019.01003

**Published:** 2019-10-24

**Authors:** Fatuma Ali Mzingirwa, Francesca Stomeo, Boaz Kaunda-Arara, Judith Nyunja, Fidalis D. N. Mujibi

**Affiliations:** ^1^Department of Fisheries, Kenya, Marine and Fisheries Research Institute, Mombasa, Kenya; ^2^Biosciences Eastern and Central Africa-International Livestock Research Institute (BecA-ILRI) Hub, Nairobi, Kenya; ^3^Department of Fisheries and Aquatic Sciences, University of Eldoret, Eldoret, Kenya; ^4^Kenya Wildlife Service, Mombasa, Kenya; ^5^Genomics Division, USOMI Limited, Nairobi, Kenya

**Keywords:** *Lethrinus mahsena*, marine-protected area, genotyping by sequencing, genetic connectivity, DArT markers

## Abstract

Marine-protected areas (MPAs) have the potential to enhance fisheries through transport of larvae or by a net emigration of adult and juvenile fish to adjacent fished areas. A network of appropriately located MPAs will have the potential to reseed fished areas and other MPAs. Connectivity studies are therefore important to assess the effectiveness of a network of MPAs and to determine the spatial scale necessary for spillover effects. The principal aim of this study was to determine the potential for Kenyan MPAs to reseed adjacent fishing zones by evaluating the levels of genetic differentiation of populations of *Lethrinus mahsena*, a commercially important fish, along a continuum of protected and nonprotected sites. Fish samples were collected from MPAs (Mombasa and Kisite Mpunguti Marine Parks) and the fished reserves adjacent to the two MPAs. Total length and weight of the fish from the sites and fin clips from one of the pectoral fins were collected and preserved in 90% ethanol. Genomic profiles for each sample were obtained through genotyping by sequencing using diversity array technology markers. Results from population structure, diversity, and admixture analyses indicated very low genetic differentiation (*F*_ST_ = 0.00184, *P* > 0.05) and low population substructure between samples obtained from the study locations implying a free exchange of fish across protected and nonprotected sites. There was a high gene flow and multidirectional migration rate among the sampling sites. Inbreeding was moderately high (*F*_IS_ = 0.15, *P* < 0.05) in the marine parks, indicating high relatedness and probably limited mating options for the species due to small population size or spatial restriction. The lack of genetic differentiation between protected areas and open fishing grounds is indicative of genetic connectivity for the sky emperor. This reinforces the significance of maintaining protected areas to serve as breeding and spawning grounds of fish without adversely affecting the livelihoods of communities that depend on the various fisheries linked to MPAs.

## Introduction

Establishment of marine-protected areas (MPAs) has been one of the most popular approaches used to protect oceans from anthropogenic threats (Steffen et al., 2007). MPAs have also been advocated worldwide as a powerful tool for conservation and management of fisheries and marine ecosystems (Pujolar et al., 2013). One of the major benefits of MPAs is to safeguard the populations of target species in order to benefit fisheries by means of spillover of adults and enhanced larval dispersal (Claudet et al., 2008).

Patterns of population connectivity in a network of protected areas are important in understanding the supply of adults and larvae into and out of a reserve (Lubchenco et al., 2003; Roberts, 2012). Genetic connectivity considerations and likely influence of physical and biotic factors play an important role in the design of MPAs (Palumbi, 2003). These areas are also developed based on the assumption that they will act as sources of recruits and that there will be sufficient dispersal among them to maintain connectivity (Palumbi, 2003; Shanks et al., 2003). There are certain marine ecosystems, such as tropical reefs, which have high levels of connectivity among different populations (Ayre and Hughes, 2000). Nonetheless, there is a general paucity of empirical data underpinning connectivity within and among MPAs worldwide (Botsford et al., 2001; Palumbi, 2003).

The operations and effectiveness of marine reserves differ based on their intended goals. However, many reserves are intended to benefit an ecosystem on a scale larger than their boundaries (Agardy, 1994). In particular, there are fishery reserves, whose economic value depends on the export of individual members into regions where fishing is allowed (De Martini, 1993). Studies have shown that isolated reserves may help build up a population of spawning adults for an overfished species. In such cases, the movement of these individuals from the reserves to the designated fishing zones is expected (McClanahan and Kaunda-Arara, 1996; Kaunda-Arara and Rose, 2003). Although there is sufficient information about the positive effects of marine reserves on the size and abundance of heavily fished species within the boundaries of a reserve (Roberts, 1997; Palumbi, 2001; Halpern, 2003), there are relatively few studies on population connectivity across reserves through exports. Empirical studies of reserve export have shown that fish tagged within reserves have been captured outside the protected zones (Attwood and Bennett, 1995; Kaunda-Arara and Rose, 2004), and fisheries yields have increased outside of the reserve boundaries (McClanahan and Kaunda-Arara, 1996).

There is a poor understanding of the interactions between dispersal and oceanic features; hence, the measurement of connectivity and consequently, the design of MPAs, and their networks remain an extremely difficult challenge (Green et al., 2014). Further, despite the evidence of gene flow among reefs, the level of genetic differentiation of fish populations within and between the reefs is not well understood or characterized (Palumbi, 2003). It is in this context that genetic tools have the potential to estimate the rate of exchange among populations and provide a measure of connectivity, which can help determine the appropriate spatial scale at which effective single MPAs and/or MPA networks should be designed (Grorud-Colvert et al., 2014).

In this study, the sky emperor *Lethrinus mahsena* was used as a model species to study the genetic connectivity of reefs in coastal Kenya. This species is widely distributed in the Indian Ocean, the red sea, the east coast of Africa, and Sri Lanka and has also been recorded in Southern Japan and Polynesia (Froese and Pauly, 1998). *L. mahsena* occupies shallow habitats ranging from 2 to 100 m in depth and has been observed predominantly in coral reefs or adjacent seagrass areas (Carpenter and Allen, 1989). Like the majority of lethrinids, this species is relatively long‐lived (up to 27 years) and is a protogynous hermaphrodite (Grandcourt, 2002; Ebisawa, 2006) with a dispersive pelagic larval stage (Nakamura et al., 2010).

There are four marine parks (no-take areas) in Kenya covering about 25% of the coastline and spanning from south to north of the coast. Genetic connectivity of fish populations across the MPAs has not been quantified, although large-scale movements of adults across MPAs are known to occur (e.g., Kaunda-Arara and Rose, 2003). The extent to which these movements influence genetic differentiation among populations is important for the function of the reserves at a metapopulation level. This study therefore aimed at assessing the level of connectivity between MPAs and open fishing zones in Kenya by characterizing the genetic differentiation and substructure populations of *L. mahsena.* The sky emperor is an economically important species in coastal East Africa and is heavily fished. The heavy fishing pressure on the populations likely creates distinct populations on the reefs, making it a suitable candidate for examining the connectivity of populations across a gradient of exploitation.

## Materials and Methods

### Study Sites

**Figure 1:**
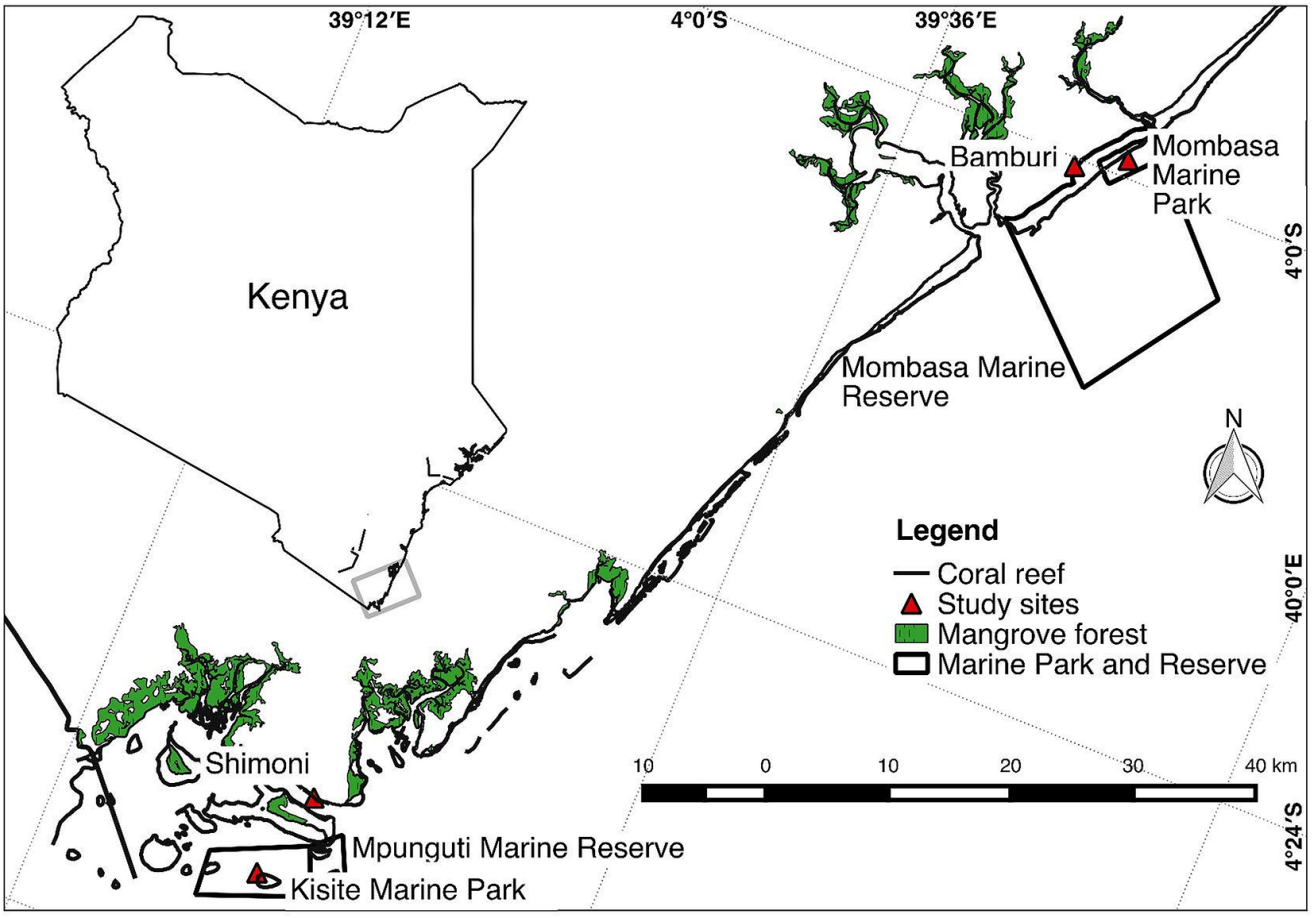
Map of a section of the Kenyan coast showing where sampling of *Lethrinus mahsena* was conducted. Bamburi comprises samples from Mombasa marine reserve and neighboring fishing areas, while Shimoni comprises samples from Mpunguti marine reserve and its adjacent fishing areas.

The study was carried out on four reef sites (Mombasa Marine Park [MMP], Mombasa Marine Reserve, Kisite Marine Park, and Mpunguti Marine Reserve) of different protection levels in coastal Kenya (**Figure 1**). Two of the sites (MMP and Kisite Marine Park) are protected and exclude extractive exploitation of resources and are designated as “marine parks.” Mombasa and Mpunguti Marine Reserves are buffer areas adjacent to the parks where regulated fishing by “traditional” methods is practiced. MMP and Mombasa Marine Reserve were established in 1986. They have a size of 10 and 200 km^2^, respectively, while Kisite Mpunguti Marine Park and its reserve were established in 1978 and have a size of 28 and 11 km^2^, respectively. The open access sites have no formal regulatory framework. Sampling was done at the protected sites (MMP and Kisite Marine Park), the adjacent marine reserves (Bamburi adjacent to MMP and Mpunguti reserves in Shimoni), and nonprotected reef sites away from the parks (**Figure 1**).

Kenyan coral reefs are predominantly shallow (10–12 m at high tide) lagoonal fringing reefs that run parallel to the coastline and have a mosaic of substrate (seagrass beds, benthic algae, sand, coral rubble, live and dead corals) characteristics. These lagoonal reefs have grossly comparable habitat and substrates type along the coast (Kaunda-Arara and Rose, 2004). The Coast experiences seasonality caused by both northeasterly and southeasterly monsoon winds described in details in McClanahan (1988). Briefly, the northeast monsoon season (November–March) is a period of calm seas, elevated sea surface temperatures, and higher salinities, while the southeast monsoon season (April–October) is characterized by rough seas, cool weather, lower salinities, and higher primary productivity.

### Sample Collection

Collection of samples from the MMP was done using local traps. Five traps were deployed to catch fish on each sampling day. Prior to deployment, the traps were first baited with benthic algae and mashed tissue of mangrove gastropod. Deployment of the traps was done by a motorboat at low tide and retrieved during the subsequent low tide on the following day. During retrieval, traps were hauled up and *L. mahsena* sorted out from the catch and measurements on weight (g) and total length (cm) taken. Fin clips were obtained from pectoral fins and preserved in 1.5-mL vials of absolute ethanol for laboratory analysis. After measurements and sample collection, all fish were returned to the capture sites. The traps were then cleaned, baited, and redeployed.

In the reserves and fished sites, samples of *L. mahsena* were obtained from artisanal fishermen that fish these areas. Measurements and fin clip samples were then collected as outlined above before fish were given back to the fishers.

Unfortunately, in Kisite Marine Park, no specimens of *L. mahsena* were collected even after several attempts, so this allowed only three sites of sample collection, namely, MMP, Bamburi (comprising of samples from Mombasa Marine Reserve), and Shimoni (which comprised samples from Mpunguti Marine Reserve and the fished sites). We were not able to distinguish between samples from the reserves (Mombasa and Mpunguti) and open fishing grounds because we relied on samples from the fishermen who were not equipped with GPRS for geographical positioning. Sample size from each site is presented in **Table 1**.

**Table 1:** Description of sampling location and number of samples collected and genotyped from Mombasa Marine Park, Bamburi, and Shimoni fishing grounds in coastal Kenya.

Site	Geographical coordinates	Sample size	Genotyped samples
Mombasa Marine Park	−4.02768S, 39.7395E	30	24
Bamburi	−3.99666S, 39.71887E	100	33
Shimoni	−4.64717S, 39.38041E	100	37

All molecular sample analyses were done at the Biosciences eastern and central Africa–International Livestock Research Institute Hub (BecA-ILRI Hub), Nairobi, Kenya, following storage at −20°C.

### DNA Extraction

Genomic DNA was extracted from the ethanol preserved fin clips using DNeasy blood and Tissue kit (Qiagen, Germany) following the manufacturers’ protocol. DNA integrity was assessed by 1% agarose gel electrophoresis, stained with GelRed. DNA quality was checked using Nanodrop spectrophotometry (Thermo Fisher Scientific, Waltham, MA, USA), while quantitation was done using the Qubit® fluorometer 2.0 (Thermo Fisher Scientific). Samples were diluted to 100 ng/µL with 1× Tris-EDTA (TE) buffer. Due to limited resources available for the genotyping, a random batch of 94 samples was selected for the study, taking into account stratification based on sampling site (**Table 1**).

### Diversity Array Technology Genotyping

Diversity array technology (DArT) reveals DNA polymorphism by querying representations of genomic DNA samples for the presence/absence of individual fragments. The principle of DArT genotyping focuses on a metagenome by capturing allelic diversity of the organism of interest in order to limit the potential for ascertainment bias; this is a bias introduced from using markers developed from a small sample of the genotypes that are being studied (Kilian et al., 2012).

In the present study, DNA samples were sent to Diversity Array Technology Pty Ltd, Canberra, Australia (http://www.diversityarrays.com/dart-map-sequences), for whole-genome scan using DArT markers. This protocol mainly works by three major processes: restriction enzymes (REs) digestion, adapter ligation, and amplification of adapter-ligated fragments. The main reason for choosing RE-based methods over other methods is the high level of precision (selectivity and reproducibility) of REs (Kilian et al., 2012).

DArT sequencing libraries were constructed in a 94 plex. DNA samples were processed indigestion/ligation reactions as per the procedures outlined in Kilian et al. (2012). Briefly, 100 ng/µL of purified genomic DNA was codigested with REs; a combination of a rare cutter, Pst1 (Poland et al., 2012), and a frequent cutter, *Sph*I (Poland et al., 2012; Nguyen et al., 2018), was applied to digest the DNA. Two compatible adaptors corresponding to the two RE overhangs were used. The *Pst*I-compatible adapter was designed to include Illumina flow-cell attachment sequence, sequencing primer sequence, and a “staggered” varying length barcode region. The reverse adapter contained flow cell attachment region and *Sph*I compatible overhang sequence. Only “mixed fragments” (*Pst*I-*Sph*I) were effectively amplified in 30 rounds of polymerase chain reaction (PCR) using the following reaction conditions: 94°C for 1 min, then 30 cycles of 94°C for 20 s, 58°C for 30 s, 72°C for 45 s, and 72°C for 7 min (Nguyen et al., 2018). This was immediately followed by applying equimolar amounts of amplification products from each sample to c-Bot (Illumina) bridge PCR. The PCR products were finally sequenced on Illumina Hiseq 2500. The sequencing was run for 77 cycles.

### Data Filtering and Single-Nucleotide Polymorphism Calling

Sequences generated were processed using proprietary DArT analytical pipelines (PLs), which consisted of a primary and a secondary PL. In the primary PL, the Fastq files were processed to filter away poor-quality sequences, such as those with reproducibility below 90% and read depth lower than 3.5 for single-nucleotide polymorphisms (SNPs). A more stringent selection criterion was applied to the barcode region compared to the rest of the sequence. This provided the assignments of the sequences to specific samples carried in the “barcode split” step and was very reliable. Only one sample was dropped due to low coverage across loci, but individual sequences were removed since they did not meet the above criteria. Approximately 1.2 million sequences per barcode/sample were identified and used in marker calling.

For SNP calling, identical sequences were collapsed into “fastqcoll files” in DArTSoft14. The fastqcoll files were “groomed” using DArT PL’s proprietary algorithm, which corrects low-quality base from singleton tag into a correct base using collapsed tags with multiple members as a template. The “groomed” fastqcoll files were used in the secondary PL for DArT PL’s proprietary SNP and Silico DArT (presence/absence of restriction fragments in representation) calling algorithms. Multiple samples were processed from DNA to allelic calls as technical replicates, and scoring consistency was used as the main selection criterion for high-quality/low error rate markers.

### Genotyping by Sequencing Data Analysis

#### Initial Data Sorting and Cleaning

A total of 12,383 SNP markers were subjected to data quality checks using PLINK version 1.9 (Purcell et al., 2007). Data quality control included removal of SNPs with less than 90% call rate, less than 5% minor allele frequency, and samples with more than 10% missing genotypes.

### Statistical Analysis

Genetic diversity measures such as number of private alleles and linkage disequilibrium were estimated in GENEPOP v 4.2 (Raymond and Rousset, 1995; Rousset, 2008). Overall expected heterozygosity (*H*_E_), observed heterozygosity (*H*_O_), and % number of polymorphic sites were determined using ARLEQUIN (Excoffier et al., 2005). Analyses of molecular variance (AMOVA) (Excoffier et al., 1992), genetic variation among all the individuals, pairwise genetic distances, and estimates of population structure (coefficient of *F*_ST_ and *F*_IS_) among the three sites were performed in ARLEQUIN (Excoffier et al., 2005).

Isolation by distance (IBD; Wright, 1943) among the populations was tested using Mantel procedure in ARLEQUIN version 3.5 using Euclidean geographic distance between sites. The distance matrix was obtained from ArcMap v10.3.1 (ESRI, Redlands, CA, USA).

ADMIXTURE 1.3 (Alexander et al., 2009) was used to estimate genetic structure based on maximum likelihood estimation. The identification of the best value for *K* was done by masking or holding out a subset of genotype data and then predicting those masked genotypes. ADMIXTURE was selected for this analysis because it takes much less time to compute *K* compared to similar programs like STRUCTURE (Alexander and Lange, 2011; Liu et al., 2013).

Principal component analysis (PCA) was performed to assess and visualize genetic distance and relatedness between populations. This method focuses on the spectral decomposition of a variance–covariance matrix for dimensionality reduction. Eigenvalues and eigenvectors are important for underlying population structure identification. The eigenvectors present the linear combination of the covariates, which in turn serve as the new dimensions. All the dimensions are orthogonal to each other. These linear combinations are known as the principal components. If there is an underlying structure among populations, PCA tends to separate them based on the principal components (Liu et al., 2013). The plots of the first two resulting principal components were generated in GENESIS 2.6.0.

The program BAYESASS version 3.0.3 (Wilson and Rannala, 2003) was used to examine contemporary gene flow (over the last few generations) and migration rates. BAYESASS uses a Bayesian method with Markov chain Monte Carlo algorithm and estimates the migration rate (*m*Bay) by identifying the population-specific inbreeding coefficients and genotypic disequilibrium. The program estimates the migrant ancestries of each individual and infers the migration rate by assignment method. The migrant ancestor is estimated using each individual’s genotype and the genotype frequency distributions of each population. An individual is considered migrant when the assigned ancestor differs from the population sampled. We used 5,000,000 iterations with a burn-in of 500,000 iterations and a sampling frequency of 100. The source and sink populations are essentially identified based on the differences between emigration and immigration, with the source population being a net exporter of individuals. To identify potential sources, we calculated the net immigration rate (net immigration rate = immigration rate) using a measure of the degree to which a population is a donor or a recipient of migrants (Hänfling and Weetman, 2006). The net immigration rate was calculated between all populations and subsequently averaged.

## Results

### Summary Statistics

A total of 230 fish were sampled from all sites, and sizes ranged between 17.5 and 40.6 cm (TL) for fish collected in the nonprotected sites and 17 to 35 cm (TL) for fish collected in the protected sites.

One sample did not meet the quality thresholds during SNP calling and was therefore removed from the final dataset; therefore, only 93 samples were available for further analysis. A total of 4,050 SNPs did not fit the inclusion criteria and were discarded, leaving 8,333 SNPs for further analysis. In total 774,969 data points were used for analysis. The average genotyping rate in the remaining samples was 0.9926. In Arlequin, nonpolymorphic loci were filtered and removed from the dataset; hence, we remained only 3,413 SNPs, which were used for downstream analysis. *H*_O_ was estimated at 0.27556 ± 0.13338 for the combined localities. *H*_E_ was estimated at 0.30319 ± 0.13136 (combined localities) and ranged between 0.30209 ± 0.13843 (Shi) and 0.30450 ± 0.14073 (MMP) and 0.30156 ± 0.13957. Percentage number of private alleles was 10.81% in all the localities.

### Genetic Differentiation

AMOVA among the 93 samples indicated that a nonsignificant value of 0.18% (*P* > 0.05) of the variance was due to genetic differentiation among the populations; 9.0% (*P* < 0.05) of the variance was accounted for by genetic differentiation among individuals within populations, while the remaining 90.8% (*P* < 0.05) of the variance was due to the differences within individuals (**Table 2**). The average *F*_ST_ value among the populations was 0.0018 (*P* > 0.05), indicating insignificant genetic differentiation between them.

**Table 2:** Analysis of molecular variance (AMOVA) showing genetic differentiation of 93 samples collected from Mombasa Marine Park, Bamburi, and Shimoni fishing grounds in coastal Kenya using DArT markers.

Source of variation	df	Sum of squares	Variance components	Percentage variation	P
Among populations	2	1,241.763	0.93448	0.18041	>0.05
Among individuals within populations	90	50,742.74	46.78047	9.03164	<0.05
Within individuals	92	43,733	470.2473	90.78795	<0.05
**Total**	184	95,717.51	517.9623		

Pairwise genetic differentiations between samples from fished sites (Bamburi and Shimoni) were significantly different (*P* < 0.05). On the contrary, there was no significant differentiation between samples obtained from the protected sites (MMP) and the two fished sites (Bamburi and Shimoni) (**Table 3**). Differentiation may have been caused by the presence of IBD (*P* < 0.05).

**Table 3:** Table of pairwise *F*_ST_ (*P* value in brackets) showing significant genetic differentiation between samples from Shimoni and Bamburi.

	Shimoni	MMP	Bamburi
Shimoni	*		
MMP	0.00203 (0.25225 ± 0.0503)	*	
Bamburi	0.00233 **(0.01802** ± **0.0121)**	0.00070 (0.82883 ± 0.0446)	*

*P<0.05.

### Inbreeding

Inbreeding levels were moderately high, with an average value of 0.09 (*P* < 0.05) across the study sites. The inbreeding coefficient estimates were slightly higher for samples from MMP (0.15) compared to Shimoni (0.08) and Bamburi (0.06) reserve sites as shown in **Table 3**. The differences between inbreeding coefficient values in the three sites, Mombasa, Bamburi, and Shimoni, were statistically significant (*P* < 0.05) (**Table 4**).

**Table 4:** Table of specific *F*_IS_ indices in each site revealing significant levels of inbreeding of *Lethrinus mahsena* population collected from Mombasa Marine Park, Bamburi, and Shimoni, coastal Kenya, as revealed by DArT markers.

Name of site	*F* *_IS_*	P
Shimoni	0.08	<0.0010
Mombasa marine park	0.15	<0.0001
Bamburi	0.06	<0.0245

FIS = coefficient of inbreeding.

### Population Structure Analyses

#### Principal Component Analysis

The results from the PCA yielded three clusters with a complete mixture of samples from all collection sites. Only one cluster had almost 80% of members drawn from Shimoni collection site (**Figure 3**). The extent of genetic variation accounted for by the first three principal components was low (25%), with PC1, PC2, and PC3 accounting for 10%, 8%, and 7%, respectively, of the total variation.

#### Admixture Analysis

**Figure 2:**
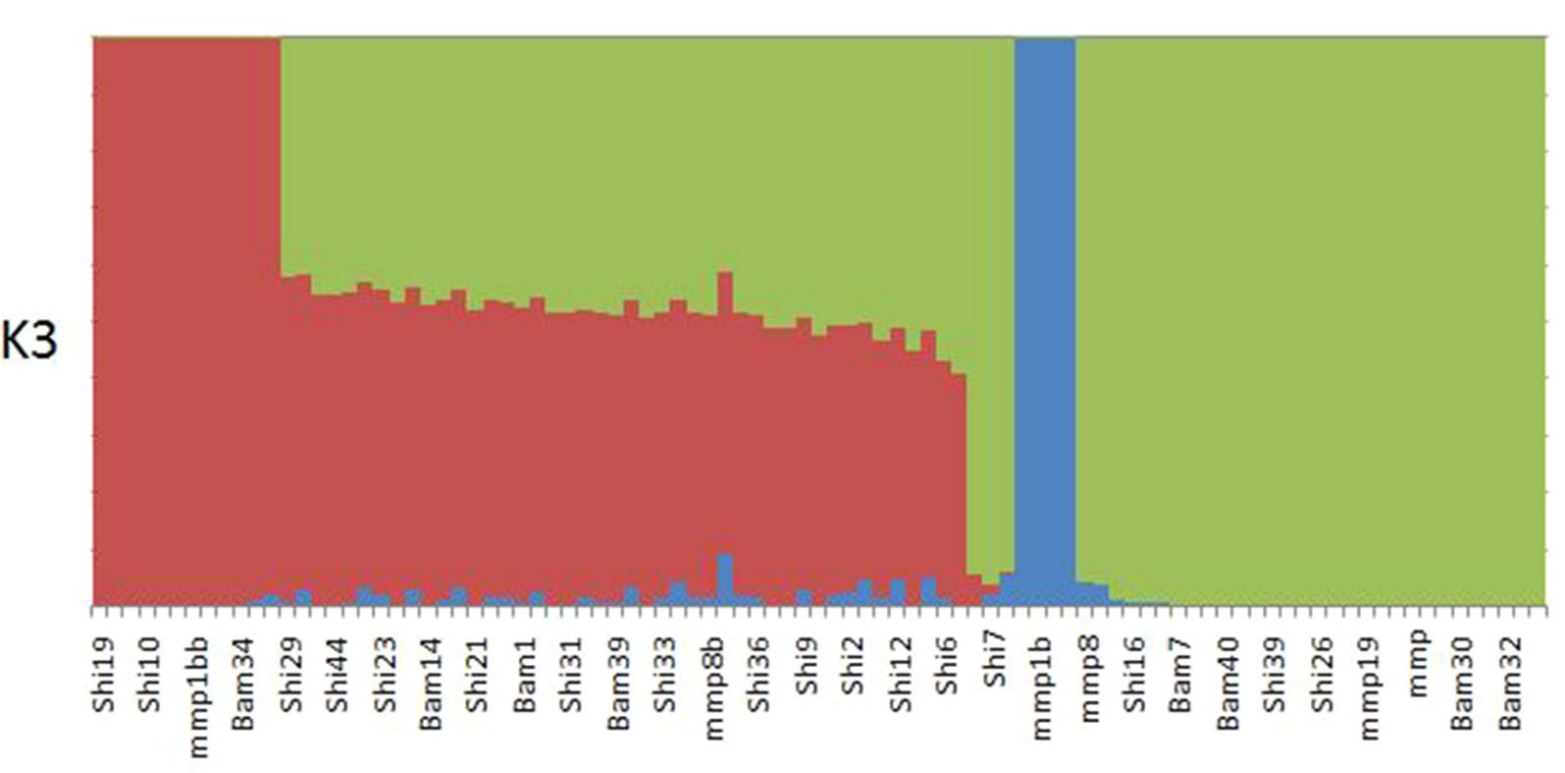
ADMIXTURE plot for K3 showing structuring of samples collected from Bamburi (Bam), Shimoni (Shi), and Mombasa Marine Park (MMP) in coastal Kenya; colors represent different populations.

**Figure 3:**
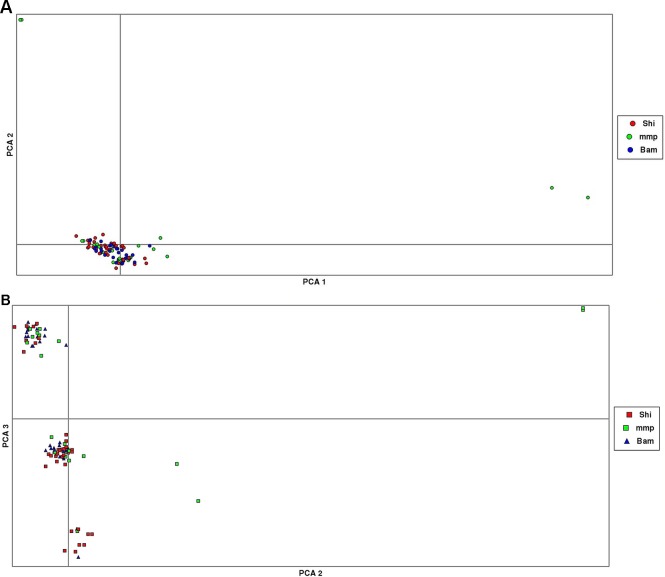
Principal component analysis (PCA) plots showing clustering of samples collected from Mombasa Marine Park (MMP), Shimoni (Shi), and Bamburi (Bam). **(A)** (PCA1, PCA2) showing one major cluster grouping samples from all localities and two minor clusters, which consists of samples from the marine park only; **(B)**, PCA (PCA2, PCA3) showing three clusters consisting of samples from all localities.

ADMIXTURE results yielded support for *K* = 1 as presented by the cross-validation (CV) error plot (**Supplementary Figure 1**) In this study, the CV errors continued to decrease as *K* increased in value, giving no clear indication of the appropriate *K* for the study population. As such, conclusions on population substructuring were based solely on visual inspection of the PCA plots. We therefore reran ADMIXTURE as a supervised analysis with *K* = 3 (the number of study sites) as the appropriate population number for the dataset (**Figure 2**). The admixture profile obtained from this supervised analysis yielded cluster profiles concordant with the PCA analysis (**Figures 3A, B**)

PCA yielded clustering of samples similar to the admixture results of K3 as shown in **Figures 3A and B**.

### Migration Rates and Gene Flow Analysis

The contemporary migration rates (*m*Bay) ranged from 0.008 to 0.25, and the majority of the estimates had 95% confidence intervals that were different from zero, indicating substantial recent migration among the sites. There was minimal migration flow from either MMP to Bamburi and Shimoni (**Table 5**). Bamburi exhibited significant out-migrations to the other sites and a positive net migration rate. There was no negative emigration; hence, no site was considered as a source for juvenile/adult fishes. Therefore, there was multidirectional migration among the sites. However, fewer migrants originated from MMP contrary to the notion that these parks should seed fished sites.

**Table 5:** Posterior means of contemporary migration rates estimated by BAYESASS (values in brackets are standard deviations, and mean values more than 0.1 are considered significant).

Population	Migration Rates
Bam→MMP	0.1452 (0.0337)
Bam→SHI	0.2500 (0.0225)
MMP→BAM	0.0095 (0.0092)
MMP→SHI	0.0084 (0.0082)
SHI→BAM	0.1713 (0.0278)
SHI→MMP	0.1735 (0.0340)

## Discussion

### Low Genetic Differentiation and High Connectivity

The results demonstrate nonsignificant (*P* > 0.05) and low genetic differentiation among the study sites. Low genetic differentiation could be caused by lack of population isolation, variance in individual reproductive success, and possible mixing of larvae from genetically different sources (Soule and Simberloff, 1986). Significant genetic differentiation was, however, detected in samples between Shimoni and Bamburi fished sites, although using Mantel (1967) procedure, the sites were statistically significant, meaning the differentiation was likely only due to geographical distances. Lack of genetic differentiation may indicate existence of fish spillover as evidenced by previous studies (McClanahan and Kaunda-Arara, 1996; Kaunda-Arara and Rose, 2003; Kaunda-Arara and Rose, 2004) and suggests that self-recruitment and local larval retention in the species may be limited as is the case in most coral reef fish with long larval duration (Leis, 1991).

It is important to use a combination of methods for estimating genetic differentiation. *F* statistics estimates assume that the current subpopulations or populations were derived from a common ancestral population (or subpopulation) that was in Hardy–Weinberg equilibrium and in linkage equilibrium (Edwards and Beerli, 2000). In our study, although a significant level of differentiation estimated by the *F*_ST_ value was obtained, there should be a correlation based on other analysis such as PCA, and ADMIXTURE to give strong support for real differentiation. However, we favored outputs of AMOVA because it is considered a powerful tool and is widely used compared to other methods of estimating genetic differentiation, hence providing a basis for comparison (Meirmans and Liu, 2018). AMOVA is based on distance matrix and in-cooperates more genetic information into the analysis; hence, it is viewed superior to allele-based methods (Nievergelt et al., 2007; Meirmans and Liu, 2018). We did not base our genetic differentiation arguments on the pairwise genetic differentiation because there was no population structuring. The Mantel test further confirmed that genetic differentiation was caused by geographical distance (Bird et al., 2011), as the distance between Bamburi and Shimoni was furthest among the sites.

The results indicate a lack of population substructuring, and despite our decision to run a supervised admixture analysis with *K* = 3 to represent the sampling sites, there was evidence of genetic connectivity between the sites. By declaring *K* = 3, we detected unique genetic backgrounds specific to each location, but with evidence of admixture in many members extant in the sampling locations. The panmictic nature of the population provides evidence that there is connectivity between MPAs and surrounding fisheries. Similar results were reported by Teske et al. (2010), who obtained a high level of connectivity in *Chrysoblephus laticeps* between marine reserves and exploited areas in South Africa. The high level of connectivity provides an adequate tool for managing overexploited reef fishes.

A lack of genetic differentiation between MMP and the adjacent Bamburi fished site also signifies the presence of free movement and migration of individuals between the two sites. Nonetheless, the lack of genetic structure revealed by *F*_ST_ and ADMIXTURE indicates the possibility of genetic connectivity of *L. mahsena* in the protected and exploited areas. This likely genetic homogeneity indicates that coral reef populations in Kenya may not require spatially explicit regulatory and management models (*sensu;* Hanski, 1991).

Genetic structuring could characterize some marine reserve populations (Bell and Okamura, 2005). The genetic diversity of coral reef fishes is expected, from ecological theory, to be less compared to species with wide ecological ranges (Charlesworth and Willis, 2009); however, studies have shown variable results on genetic diversity of coral reef fishes ranging from high (Hobbs et al., 2013) to low (Eble et al., 2009; Bay and Caley, 2011). Movements of coral reef fishes across the boundaries of marine parks (total exclusion from fishing) can affect the abundance and distribution of fishes within and outside reserves (Rakitin and Kramer, 1996; Russ and Alcala, 1996; Kramer and Chapman, 1999; Kaunda-Arara and Rose, 2004) and hence genetic differentiation between reef sites (Planes, 2002).

Several studies have reported that shallow-water species are genetically more differentiated than their counterparts in deep waters (Gaither et al., 2011), while some studies indicate that shallow-water reef fishes have high site fidelity and move only short distances as adults (Meyer et al., 2000; Friedlander et al., 2002), likely leading to genetic differentiation.

Low genetic differentiation has been observed on *Acanthurus leucosternon* in Eastern Africa based on mitochondrial DNA and *C. laticeps* and *Caffrogobius caffer, *in South Africa (Neethling et al., 2008; Teske et al., 2010; Otwoma et al., 2018). Contrary results have been observed on fish of the same family, *L. mahsena* and *Lethrinus harak*, in the South West Indian Ocean (SWIO), which showed significant genetic differentiation and structuring of samples from different localities (Healey et al., 2018). Muths et al. (2011) also detected significant genetic structuring of *Myripristis berndti* in the SWIO using nuclear microsatellites, while a previous study on the same species using Mt DNA showed lack of genetic differentiation and significant population connectivity (Craig et al., 2007). Consequently, there are variable reports in the literature on genetic differentiation and structuring patterns of marine fishes requiring more database on species from different biogeographical regions as contributed by this study.

### Migration Rates and Gene Flow

We found multidirectional migration of *L. mahsena* between the sites, although significant migrants came from Bamburi (marine reserve adjacent to MMP), and a few migrants originated from MMP. This is contrary to existing studies that have reported gene flow from the marine parks to fished sites (McClanahan and Kaunda-Arara, 1996; Halpern, 2003; Lester et al., 2009). Significant migration from Bamburi site could be attributed to diffuse nature of fish movements due to the absence of defined boundaries as in parks that often provide a topographic and fishing-induced barriers to movements (Gell and Robers, 2003; Kaunda-Arara and Rose, 2004). Several authors have reported that gene flow in coral reef fish populations was mainly caused by dispersal of larvae; for example, high gene flow was reported for *Scarus ghobban* in the Western Indian Ocean and was thought to be attributed to a pelagic larval phase (Visram et al., 2010), as was for *Pristipomoides filamentosus* (Gaither et al., 2011). This study contributes to the database on genetic connectivity of reef sites in the West Indian Ocean region that is necessary for management of reef fish populations.

### Higher Inbreeding in the Marine Parks

There was a moderately higher level of inbreeding of *L. mahsena* in the MMP compared to the other studied areas. It is known that small, isolated populations are at greater risk of extinction due to genetic drift, inbreeding, and the loss of adaptive heterozygote conditions (Reed, 2005). MMP (10 km^2^) is the smallest of all the sampled sites. Given that, in general, coral reef fishes do not migrate large distances, it is possible that the confinement of *L. mahsena* to a small area increases the inbreeding chances. For example, heterozygosity deficiency in the European plaice (*Pleuronectes platessa*) in the North Sea was attributed to inbreeding (Hoarau et al., 2004). Despite the Plaice having a relatively large census population size, they tend to spawn in their natal area and have high variance in reproductive success, hence increasing the probability that spawning pairs or groups were contained in related individuals due to the confined spawning area (Hoarau et al., 2004). Inbreeding could directly affect marine fish as they could fail to recover from exploitation due to lower survival and reproduction rates, lower resistance to diseases, and environmental stress (Keller and Waller, 2002) and eventually significant effect on extinction rate (O’Grady et al., 2006). Consequently, more individuals are required to maintain genetic diversity in parks calling for a review of the design criteria of MPAs in Kenya to avoid possibilities of inbreeding depressions and to include minimum viable population sizes.

In this study, we chose genotyping by sequencing (GBS) approach over other genotyping approaches. The advantage of GBS is that repetitive regions of genomes are avoided while allowing lower copy regions to be targeted with twofold to threefold higher efficiency (Elshire et al., 2011). This simplifies computationally challenging alignment problems in species with high levels of genetic diversity (Elshire et al., 2011). Given that there is no genome sequence assembled for the sky emperor, application of SNP arrays of closely related fishes would have been unsatisfactory, as would have been the use of other marker systems such as RFLP and microsatellite, because of the lower specificity or throughput, respectively (Coates et al., 2011). The DArT GBS markers were well distributed across the whole genome (Jaccoud et al., 2001). However, in our analysis, the DArT makers used showed heterozygosity deficits as was expected. DArT markers are biallelic dominant markers (Neumann et al., 2011); hence, the homozygous and heterozygous states cannot be distinguished. Despite the fact that heterozygote deficits are a common feature of many marine populations (Ayre and Hughes, 2000), the lack of ability to distinguish heterozygosity states could have been contributed to the lack of distinct cluster definition in the PCA and admixture analyses.

## Conclusion

Low genetic differentiation is probably caused by high levels of genetic connectivity of *L. mahsena* in the study locations. Active migration and high gene flow were found to occur. Significant levels of inbreeding were observed in MMP, which implies that design criteria of marine parks in Kenya need to consider the trade-offs between single large or several small reserves (Simberloff and Abele, 1982) in conserving genetic diversity and enhancing fisheries. Populations of *L. mahsena* need to be conserved at minimum viable sizes in order to increase the number of potential mating individuals so as to enhance the genetic diversity of the spawning stock biomass and resultant migratory juveniles. This study provided a first attempt to assess genetic connectivity (“genetic spillover”) and the functionality of MPAs on the Kenyan coast. A more elaborate study with larger sample sizes collected from all MPAs and better marker density is required to confirm the effectiveness of MPAs in relation to genetic diversity, connectivity, and spillover to fisheries.

## Data Availability Statement

The datasets generated for this study can be found in Figshare: https://figshare.com/s/228633c68f195fb832b7.

## Ethics Statement

This study was carried out in accordance with the recommendations of Kenya Marine and Fisheries Research Institute (KMFRI) strategic plan 2010-2015 and approved by the KMFRI scientific committee. Sampling in the marine park and other areas was conducted according to the guidelines of Kenya Wildlife Service (KWS) and KMFRI, respectively.

## Author Contributions 

FM designed and obtained funding for the study. BK-A streamlined the ideas and assisted in funding proposal writing. JN facilitated sampling in the marine parks. FM performed the molecular experiments. FDNM and FS supervised the experiments and analysis of the data. FM analyzed the data and wrote the first draft, and FDNM made suggestions and corrections. All authors read and approved the final manuscript.

## Funding

This study was supported by the International Foundation for Science (IFS) and BecA-ILRI Hub through the Africa Biosciences Challenge Fund (ABCF) program. The ABCF Program is funded by the Australian Department for Foreign Affairs and Trade (DFAT) through the BecA-CSIRO partnership, the Syngenta Foundation for Sustainable Agriculture (SFSA), the Bill & Melinda Gates Foundation (BMGF), the UK Department for International Development (DFID), and the Swedish International Development Cooperation Agency (Sida). IFS grant No: A/5716-1.

## Conflict of Interest

FDNM is a current employee of USOMI LTD. USOMI Ltd. has no commercial or financial interests in the work or outcomes of the study. The remaining authors declare that the research was conducted in the absence of any commercial or financial relationships that could be construed as a potential conflict of interest.

## Supplementary Material

Supplementary Figure 1 Cross validation (CV) error plot from Admixture analysis showing number of population of L.mahsena collected from marine protected areas and exploited fishing grounds.
