# Supplementary figures and images for: Genetic Connectivity of the Sky Emperor, Lethrinus mahsena Populations Across a Gradient of Exploitation Rates in Coastal Kenya
Source: Front Genet. 2019 Oct 24;10:1003. doi: 10.3389/fgene.2019.01003 (PMC6822600; doi:10.3389/fgene.2019.01003)

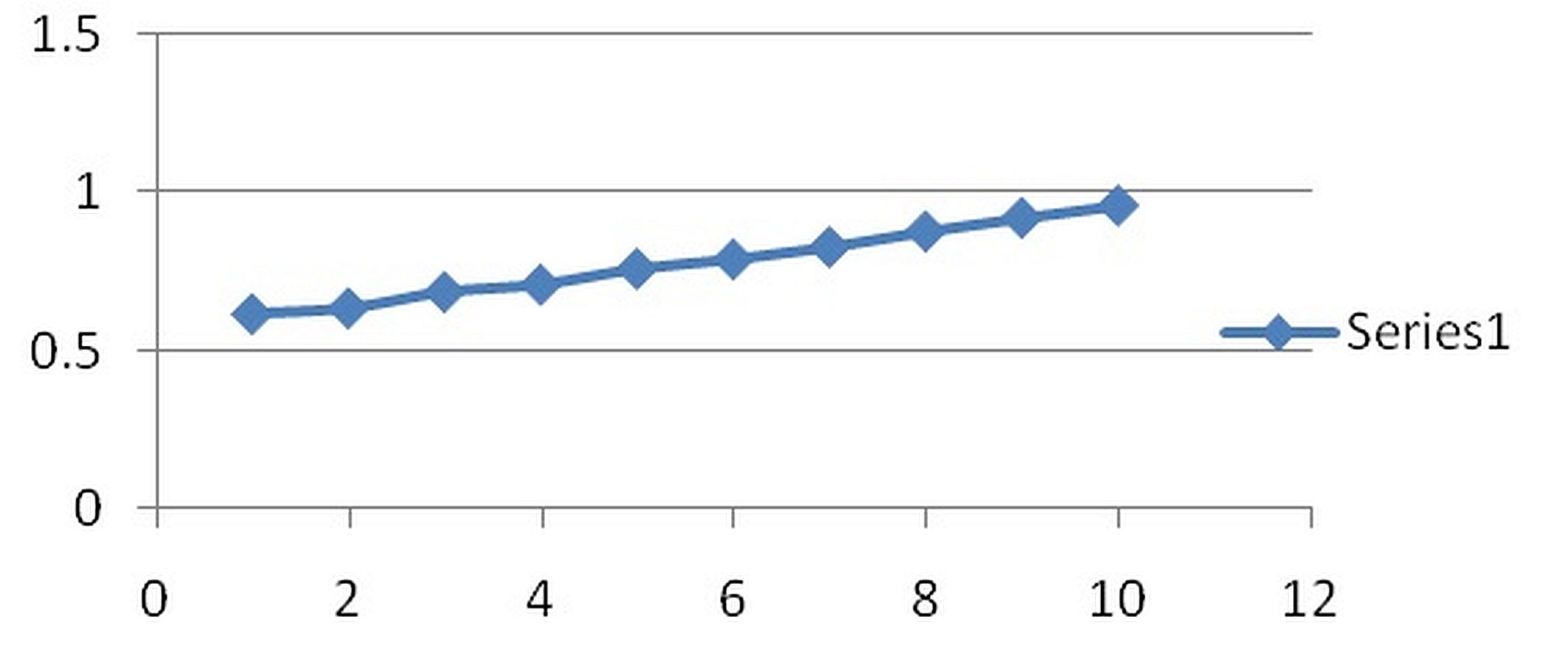

Supplement: Supplementary Figure 1 — Cross validation (CV) error plot from Admixture analysis showing number of population of L.mahsena collected from marine protected areas and exploited fishing grounds. [file Image_1.jpeg]
